# Microbiome profile of the amniotic fluid as a predictive biomarker of perinatal outcome

**DOI:** 10.1038/s41598-017-11699-8

**Published:** 2017-09-22

**Authors:** Daichi Urushiyama, Wataru Suda, Eriko Ohnishi, Ryota Araki, Chihiro Kiyoshima, Masamitsu Kurakazu, Ayako Sanui, Fusanori Yotsumoto, Masaharu Murata, Kazuki Nabeshima, Shin’ichiro Yasunaga, Shigeru Saito, Makoto Nomiyama, Masahira Hattori, Shingo Miyamoto, Kenichiro Hata

**Affiliations:** 1 Department of Maternal-Fetal Biology, National Research Institute for Child Health and Development, Tokyo, 157-8535 Japan; 2 Department of Obstetrics and Gynecology, Faculty of Medicine, Fukuoka University, Fukuoka, 814-0180 Japan; 3 Department of Computational Biology, Graduate School of Frontier Sciences, The University of Tokyo, Chiba, 277-8561 Japan; 4 Department of Microbiology and Immunology, Keio University School of Medicine, Tokyo, 160-0016 Japan; 5 Center for Maternal, Fetal and Neonatal Medicine, Fukuoka University Hospital, Fukuoka, 814-0180 Japan; 6 Department of Pathology, Fukuoka University School of Medicine and Hospital, Fukuoka, 814-0180 Japan; 7 Department of Biochemistry, Faculty of Medicine, Fukuoka University, Fukuoka, 814-0180 Japan; 8 Department of Obstetrics and Gynecology, University of Toyama, Toyama, 930-0194 Japan; 9 Department of Obstetrics and Gynecology, National Hospital Organization Saga Hospital, Saga, 849-8577 Japan; 10 Cooperative Major in Advanced Health Science, Graduate School of Advanced Science and Engineering, Waseda University, Tokyo, 169-8555 Japan

## Abstract

Chorioamnionitis (CAM), an inflammation of the foetal membranes due to infection, is associated with preterm birth and poor perinatal prognosis. The present study aimed to determine whether CAM can be diagnosed prior to delivery based on the bacterial composition of the amniotic fluid (AF). AF samples from 79 patients were classified according to placental inflammation: Stage III (n = 32), CAM; Stage II (n = 27), chorionitis; Stage 0-I (n = 20), sub-chorionitis or no neutrophil infiltration; and normal AF in early pregnancy (n = 18). Absolute quantification and sequencing of 16S rDNA showed that in Stage III, the 16S rDNA copy number was significantly higher and the α-diversity index lower than those in the other groups. In principal coordinate analysis, Stage III formed a separate cluster from Stage 0-I, normal AF, and blank. Forty samples were classified as positive for microbiomic CAM (miCAM) defined by the presence of 11 bacterial species that were found to be significantly associated with CAM and some parameters of perinatal prognosis. The diagnostic accuracy for CAM according to miCAM was: sensitivity, approximately 94%, and specificity, 79–87%. Our findings indicate the possibility of predicting CAM prior to delivery based on the AF microbiome profile.

## Introduction

Preterm birth, which occurs in 5–18% of all pregnancies, is caused by multiple pathological conditions^1,2^ and is the leading factor in perinatal mortality and morbidity, and childhood neurological problems^3–5^. Intrauterine infection is linked to spontaneous preterm labour, which accounts for about two-thirds of all preterm births^1,6,7^, and a minimum of 25–40% of premature infants are born to mothers with intrauterine infection^7–9^. In 30% of intrauterine infections, bacteria are identified in the foetal circulation^1,10^, and it is known that foetal infections induce a systemic inflammatory response^11^, which is suggested to cause abnormalities in the central nervous system, especially the white matter, by epidemiologic studies and animal experiments^12–14^.

Chorioamnionitis is an inflammation of the foetal membranes (amnion and chorion) histologically diagnosed by the presence of acute inflammatory cells, such as neutrophils. In addition to being a gold standard for corroborating intrauterine infection, chorioamnionitis is associated with preterm birth and poor infant prognosis and is recognized as a risk factor for cerebral palsy and chronic lung disease^15–19^. The proposed diagnostic criteria prior to delivery^19,20^ have low prediction accuracy for chorioamnionitis and intrauterine infection, and do not help prevent prematurity and neonatal sepsis^19,21^.

The amniotic fluid (AF) is considered to be sterile; however, this is frequently not the case in preterm birth, and a low gestational age at delivery is shown to be associated with bacterial infection in the AF^8^. Various bacterial species are detected in the AF in cases of preterm birth^22–26^. However, *Ureaplasma* spp. are also observed at 16–20 weeks in cases of normal delivery^27^, and the detection rates of aerobic and anaerobic bacteria in the AF in full-term births are similar to those in preterm ones^28,29^; in addition, bacteria have been detected in umbilical cord blood and meconium^30,31^. These findings indicate that the AF is not necessarily sterile, even in normal pregnancies, and that the association between preterm deliveries and infection should be further clarified.

Molecular biology techniques have been proved effective for detecting *Ureaplasma* spp. and other bacteria difficult to identify using conventional culture methods^32^. However, to the best of our knowledge, metagenomic analysis with next-generation sequencing of the AF to verify the relationship between bacterial diversity and chorioamnionitis has not been conducted. Here, we performed absolute quantification of 16S ribosomal DNA (rDNA) copy numbers and sequencing of 16S rDNA amplified from the AF obtained by aseptic methods for comprehensive, quantitative analysis of AF microbiome. By examining the association between inflammation in the placenta and the bacterial composition of the AF, we demonstrated, for the first time, that microbial profiling of the AF can be used to diagnose chorioamnionitis with a high degree of accuracy prior to delivery, and to predict perinatal complications.

## Results

### Assessment of study subjects

Seventy-nine patients selected for the study were divided based on the stage of placental inflammation (Blanc’s classification)^33^: Stage III (n = 32), chorioamnionitis; Stage II (n = 27), chorionitis; and Stage 0-I (n = 20), sub-chorionitis or no neutrophil infiltration. AF samples collected in the early second trimester (mean ± SD: 16.1 ± 0.6 weeks of pregnancy) during the same period were used as the AF control (Normal AF; n = 18), while laboratory-grade water was used as blank control (Blank; n = 24) for DNA extraction and library preparation (Table S1). Demographic and clinical characteristics for Stage III, Stage II, and Stage 0-I patients were extracted from medical records (Table S2).

Stage III was significantly different from Stage 0-I regarding multigravida, preterm premature rupture of membranes, antibiotic use before amniocentesis, number of caesarean sections, gestational age at amniocentesis, maternal inflammation (white blood cell [WBC] count, C-reactive protein [CRP] value), WBC count in the AF, funisitis of umbilical cord, extended days of hospital stay from admission to birth, neonatal inflammation (WBC count, CRP value, IgM level, funisitis of the umbilical cord), and antibiotic use for newborns (Table S2). Between Stage II and Stage 0-I, significant differences were observed only in caesarean sections, maternal CRP value, extended days of hospital stay, and neonatal IgM value (Table S2).

### Quantification of microbial colonization

**Figure 1:**
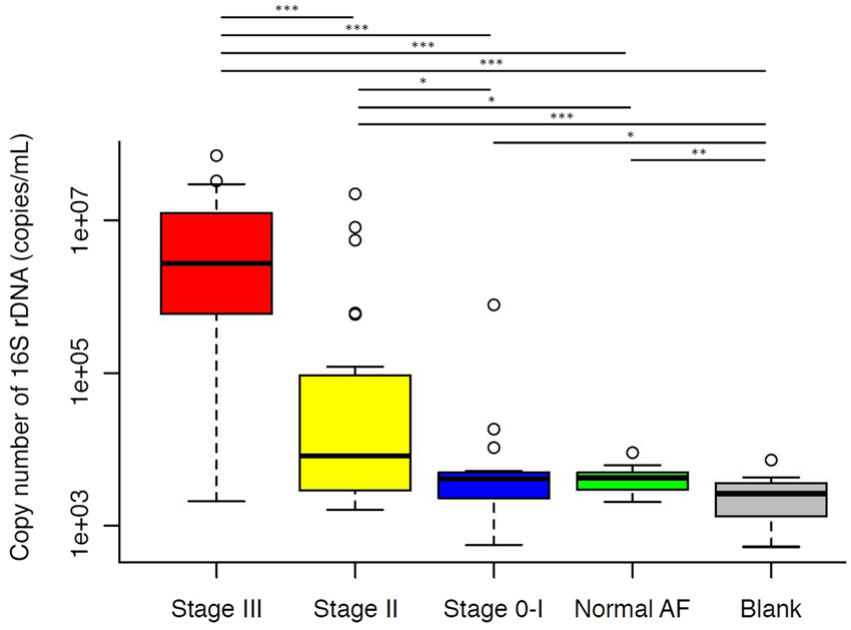
Microbial abundance in amniotic fluid samples. Microbial load was assessed based on 16S rDNA copy numbers per 1 mL AF using dPCR with universal primers 27Fmod and 338R and EvaGreen dye. The copy numbers in Stage III and Stage II were significantly higher than those in Stage 0-I/Normal AF/Blank; no differences were detected, only between Stage 0-I and Normal AF. Two-tailed probabilities were calculated by the Mann–Whitney test; *P < 0.05, **P < 0.01, ***P < 0.001.

To assess bacterial load, we determined 16S rDNA copy numbers per 1 mL of AF using digital (d)PCR with a universal primer set^34,35^ and EvaGreen dye. The median 16S rDNA copy number in Stage III was 328 and 656 times higher than that in Stage II and Stage 0-I, respectively (2.70 × 10^6^ vs. 8.25 × 10^3^ and 4.12 × 10^3^, respectively; *P* < 0.001), and that in Stage II was 2.0 times higher than that in Stage 0-I (*P* = 0.019) (Figs 1, S1).

While Stage 0-I and Normal AF demonstrated significantly higher copy numbers than Blank (*P* = 0.035, 0.003, respectively), no difference was observed between Stage 0-I and Normal AF (*P* = 0.696), indicating that the AF in the early second trimester of a normal pregnancy is not necessarily as sterile as laboratory-grade water.

### Comparison of bacterial diversity

**Figure 2:**
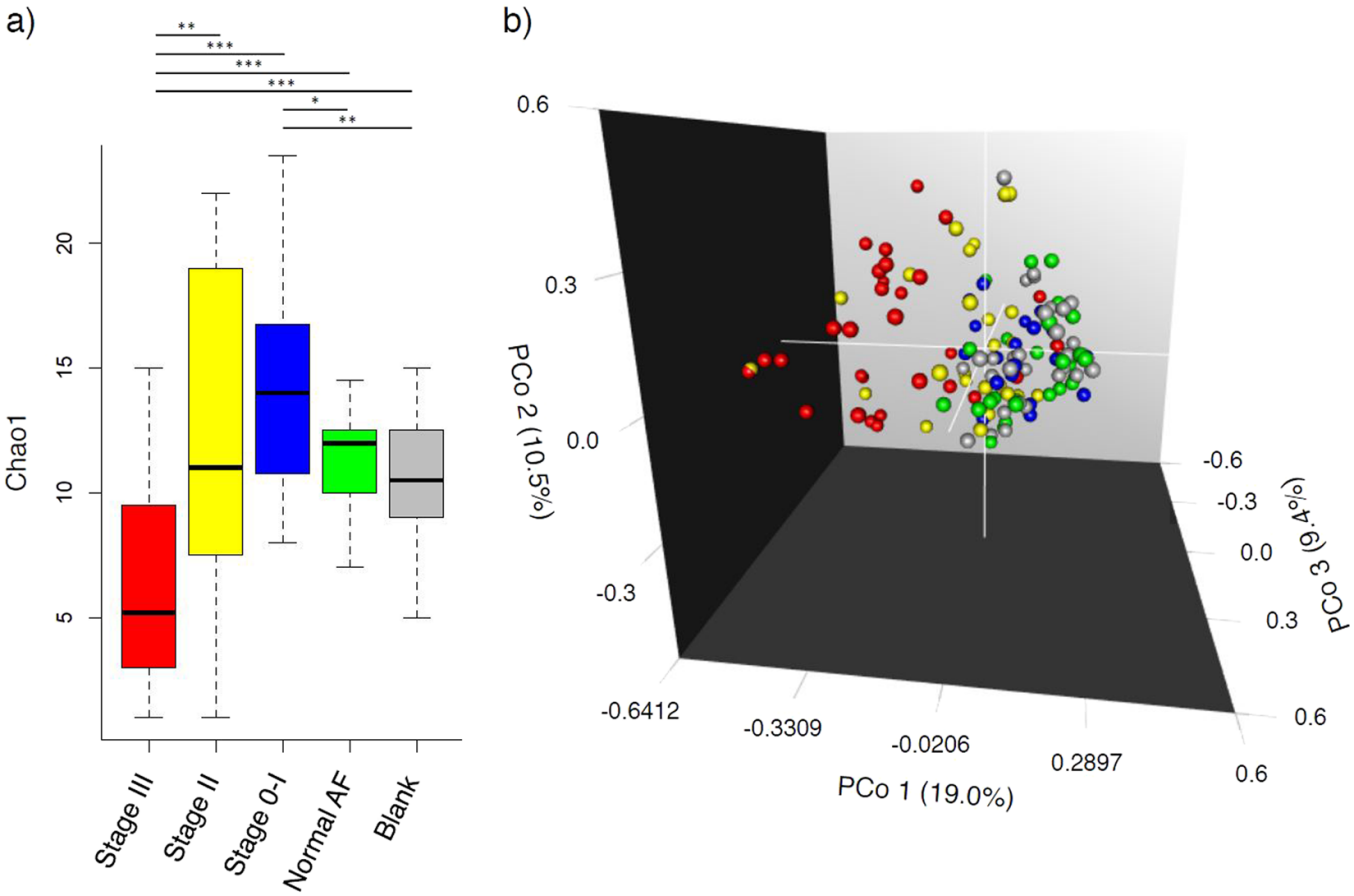
Numbers of OTUs (Chao1 index) and 3D-PCoA based on un-weighted UniFrac distances. Amplicons of 16S rDNA were sequenced using 27Fmod and 338R primers. (**a**) Sequences were clustered into OTUs with a 97% identity threshold and the α-diversity index (Chao1) was calculated for each sample. In Stage III, Chao1 was significantly lower than in the other groups. (**b**) Multidimensional composition of each group was determined based on matrix data for un-weighted UniFrac distance. Clustering of Stage III (red) samples differed from that of Stage 0-I (blue)/Normal AF (green)/Blank (grey); Stage II (yellow) was scattered between the two clusters. Three-D PCoA was performed with R; *P < 0.05, **P < 0.01, ***P < 0.001 by Mann–Whitney test.

Using the same universal primer set, we amplified and performed parallel sequencing of 16S rDNA in 97 AF samples and 24 blank controls. Only one sample (N11) did not yield sufficient reads for metagenomic analysis. Operational taxonomic units (OTUs) were created and within-community (alpha) diversity was assessed by comparing the α-diversity index (Chao1) between the groups (Fig. 2a). Interestingly, Stage III demonstrated a significantly lower Chao1 than Stage II, Stage 0-I, Normal AF, and Blank (*P* = 0.001, *P* < 0.001, *P* < 0.001, and *P* < 0.001, respectively) and Stage 0-I demonstrated a significantly higher Chao1 than Normal AF and Blank (*P* = 0.038, 0.008, respectively), while no other between-group differences were detected.

To compare phylogenetic relatedness of the microbial communities, we determined UniFrac distances between samples according to OTU data^34–36^. In principal coordinate analysis (PCoA) based on un-weighted UniFrac distances, Stage 0-I, Normal AF, and Blank clustered together, while Stage III formed a separate cluster, and Stage II was scattered between these two clusters (Fig. 2b). Analysis of un-weighted UniFrac distances with PERMANOVA revealed that Stage III and Stage II were significantly different from the other groups (Stage 0-I/Normal AF/Blank), and Stage 0-I was significantly different from Blank, but there were no differences among the other groups (Table S3). In analysis of weighted UniFrac distances with PCoA and PERMANOVA, similar to the results of un-weighted UniFrac distances, Stage III was significantly different from the other groups (Stage 0-I/Normal AF/Blank), and Stage II was scattered (Fig. S2, Table S3).

### Analysis of bacterial composition in individual samples

**Figure 3:**
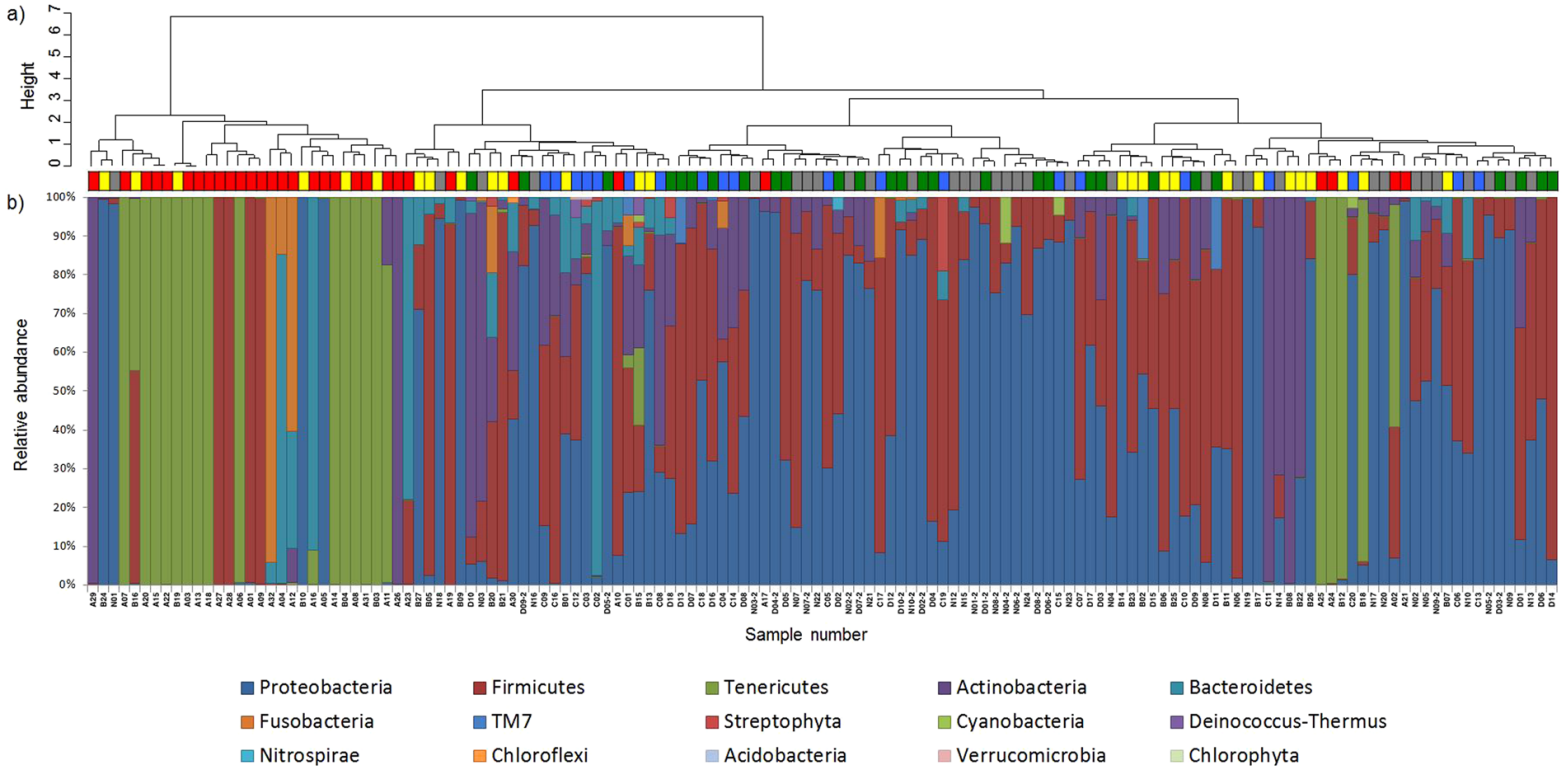
Relative abundances of different bacterial phyla in each sample. Sequences were clustered into OTUs with a 70% identity threshold and taxonomic assignments were performed by similarity searching against the standard database. The samples were rearranged by hierarchical cluster analysis using Ward’s method based on un-weighted UniFrac distances. Stage III and Stage 0-I/Normal AF/Blank formed separate clusters, while Stage II was scattered between the two clusters.

Phylum-, genus-, and species-level OTUs were created with identity thresholds of 70%, 94%, and 97%, respectively, and taxonomic structure in each OTU was assessed by similarity searching against the standard database. Sample rearrangement by hierarchical cluster analysis using Ward’s method based on un-weighted UniFrac distances (Figs 3a, S3a) mostly showed phylum-level distribution (Fig. 3). Consistent with the PCoA results, Stage III and Stage 0-I/Normal AF/Blank formed roughly separate clusters, while Stage II was scattered between the two clusters (Figs 3, S3). Consistent with the results shown in Fig. 1a, genus-level analysis indicated that multiple samples of Stage 0-I/Normal AF/Blank demonstrated a relatively high species richness, indicating complex compositions (Fig. S3). In contrast, the numbers of species in Stage III and in some samples of Stage II were extremely low (Fig. S3). Particularly noteworthy is the relative abundance of *Ureaplasma* spp. (minimum 35.5%) in fifteen samples (A2, 3, 6–8, 11, 13–15, 18, 20, 22, 24, 25, 31) of Stage III (64.3%) and six samples (B3, 4, 12, 16, 18, 19) of Stage II (22.2%) compared to all other samples (maximum 8.8%).

### Selection of bacterial species as candidate diagnostic markers

**Figure 4:**
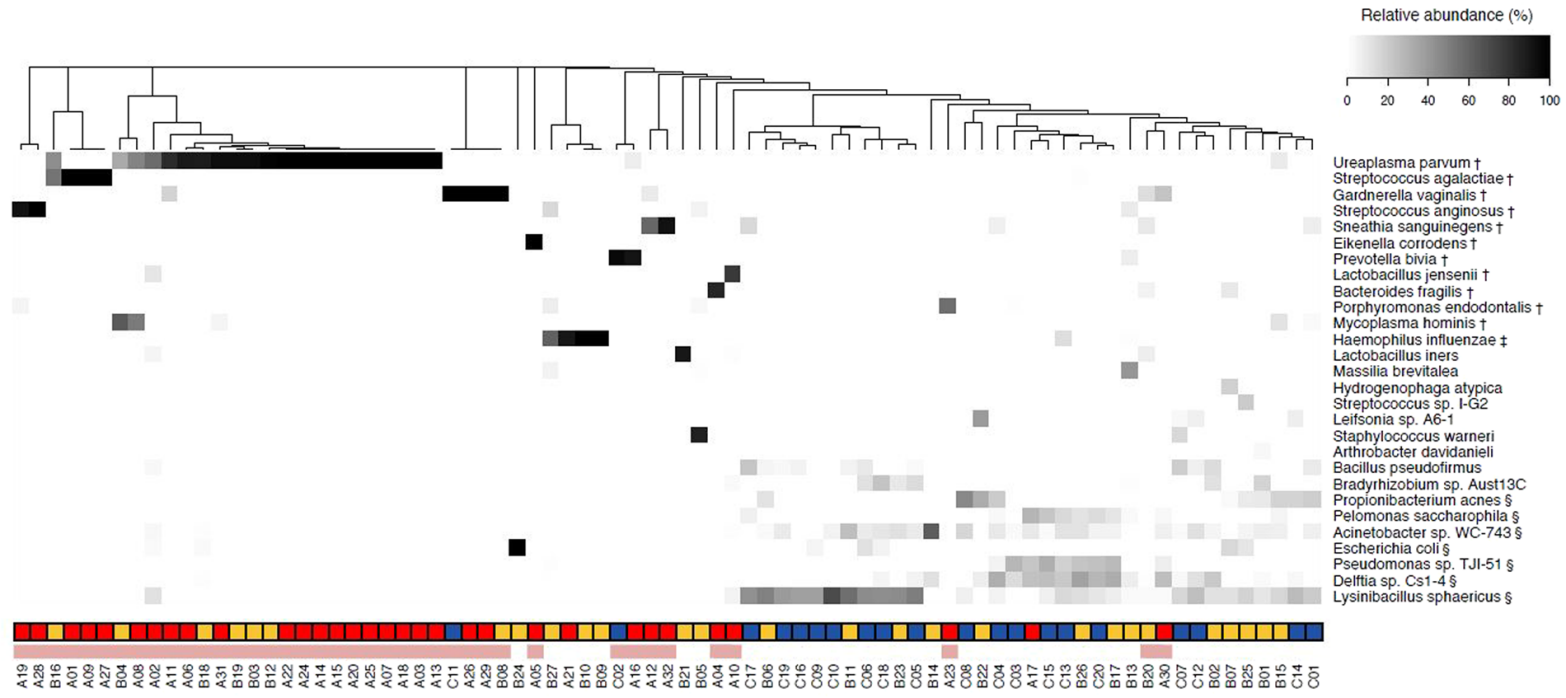
Relative abundances of the 28 most dominant species. Data on the relative abundances of the 28 most representative species were re-clustered according to the 79 samples in Stage III, Stage II and Stage 0-I. Stage III and Stage 0-I roughly formed separate clusters, while Stage II was scattered between the two clusters. The 11 most dominant species in Stage III (†) were almost non-existent in Stage0-I, while the seven most dominant species in Blank (§) were not dominant in Stage III and 40 miCAM samples (indicated by a pink bar). *H*. *influenza* (‡), which was dominant in one sample in Blank (N16), was dominant in some samples in Stage III and Stage II.

To assess the association of particular bacterial species with chorioamnionitis, the data on relative abundance of the 28 most representative species were re-clustered according to the 79 samples in Stage III, Stage II, and Stage 0-I (Fig. 4).

Stage III and Stage 0-I roughly formed separate clusters, while Stage II was scattered between the two clusters. In Stage III samples, the 12 most dominant species were *Ureaplasma parvum*, *Streptococcus agalactiae*, *Gardnerella vaginalis*, *Streptococcus anginosus*, *Sneathia sanguinegens*, *Eikenella corrodens*, *Prevotella bivia*, *Lactobacillus jensenii*, *Bacteroides fragilis*, *Porphyromonas endodontalis*, *Mycoplasma hominis*, and *Haemophilus influenzae*; however, except for *H. influenzae*, these species were nearly absent in Normal AF and Blank (maximum: 3.46%). In Blank samples, the most dominant species were *Lysinibacillus sphaericus*, *Delftia* sp. Cs1-4, *Pseudomonas* sp. TJI-51, *Escherichia coli*, *Acinetobacter* sp. WC-743, *Pelomonas saccharophila*, and *Propionibacterium acnes*, which can be attributed to inevitable very low amounts of contamination during sample preparation. *H*. *influenzae*, which was dominant in some samples of Stage III and Stage II (A21; B9, 10, 27), was also dominant in one Blank sample (N16); indicating that contamination is nearly unavoidable.

The 11 species (*U. parvum, S. agalactiae, G. vaginalis, S. anginosus, S. sanguinegens, E. corrodens, P. bivia, L. jensenii, B. fragilis, P. endodontalis, and M. hominis*) that were remarkably dominant in Stage III but not in Blank were considered as candidate markers for prenatal diagnosis of chorioamnionitis; therefore, the samples in which any of these species demonstrated the highest abundance were considered positive for microbiomic chrioamnionitis (miCAM). According to this criterion, 30 out of 32 samples in Stage III (94%), 8 out of 27 samples in Stage II (30%), and 2 out of 20 samples in Stage 0-I (10%) were miCAM-positive (Table S4). The accuracy of chorioamnionitis diagnosis (Blanc’s stage III) based on miCAM was as follows: sensitivity, 93.8%; specificity, 78.7%; positive predictive value, 75.0%; negative predictive value, 94.9%.

### Comparison of clinical characteristics between miCAM and non-miCAM samples

**Figure 5:**
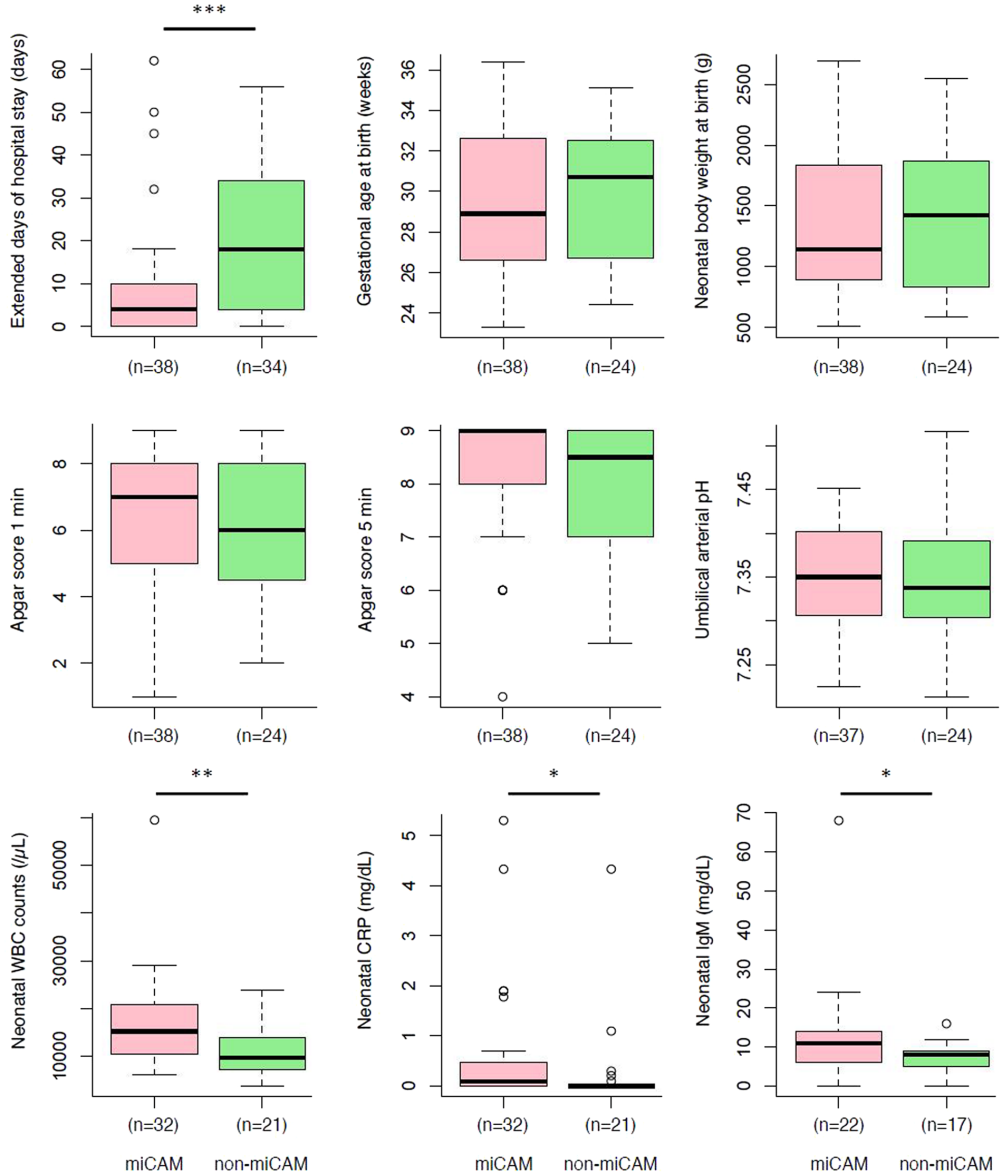
Comparison of perinatal outcomes between miCAM and non-miCAM subgroups. Comparison of continuous variables related to maternal and perinatal outcomes between miCAM and non-miCAM samples revealed that miCAM was significantly associated with many prognostic parameters of perinatal outcome. *P < 0.05, **P < 0.01, ***P < 0.001 by Mann–Whitney test.

We compared continuous variables related to maternal and infant perinatal outcomes between miCAM and non-miCAM samples (Fig. 5, Table S5). Although no significant difference was observed in gestational age and neonatal body weight at birth, Apgar score, or umbilical arterial pH; the miCAM subgroup demonstrated significantly shorter duration of maternal hospital stay from admission to birth, and higher WBC count, CRP value, and IgM levels in neonatal peripheral blood immediately after birth (Fig. 5). These results indicated a significant correlation between miCAM and adverse prognostic parameters in both mother and infant.

### Diagnostic accuracy for miCAM and chorioamnionitis assessed by clinical and metagenomic sequence data

Next, we used clinical and laboratory data, DNA concentrations, and 16S rDNA copy numbers to calculate the area under the curve (AUC), the Youden index for the receiver operating characteristic (ROC) curve, cut-off value, and diagnostic sensitivity and specificity (Table S6, Fig. S4). Compared to body temperature, heart rate, WBC count, and CRP level in maternal peripheral blood, DNA amount and especially, 16S rDNA copy number demonstrated higher diagnostic accuracy for both miCAM and chorioamnionitis. Regarding miCAM, the diagnostic accuracy according to 16S rDNA copy number with an AUC of 0.909 (asymptotic 95% confidence interval [CI]: 0.838–0.980) and a cut-off value of 1.19 × 10^4^ was: sensitivity, 94.9%, and specificity, 78.9%. For chorioamnionitis (Blanc’s stage III), the diagnostic accuracy with an AUC of 0.926 (asymptotic 95% CI: 0.868–0.985) and a cut-off value of 1.73 × 10^4^ was: sensitivity, 93.5%, and specificity, 87.0% (Table S6, Fig. S1).

## Discussion

A close relationship between intrauterine infection and preterm labour have been confirmed^22,23,26,36^, and recent studies have shown the association of placental microbiome composition with preterm birth and chorioamnionitis^37,38^. However, to the best of our knowledge, there was no report on metagenomic analysis of the AF to verify the relationship between bacterial community structure and placental inflammation. We conducted a metagenomic analysis of the AF obtained by aseptic methods from patients with or without chorioamnionitis and defined miCAM, which showed predictive utility in the identification of patients with poor prognosis regarding preterm delivery and neonatal status.

Of the 28 most dominant species identified (Fig. 4), the 12 most dominant species in Stage III (*U. parvum, S. agalactiae, G. vaginalis, S. anginosus, S. sanguinegens, E. corrodens, P. bivia, L. jensenii, B. fragilis, P. endodontalis, M. hominis, and H. influenzae*) primarily colonize the urogenital system (except *B. fragilis and P. endodontalis*, which are indigenous to the intestinal tract and oral environment, respectively); many of these may cause foetal infections such as meningitis and/or pulmonary disease, which can lead to brain dysfunction, epilepsy, hearing loss, and developmental disorders^19,39–45^.

Eleven of the 12 bacterial species dominant in Stage III (except *P. endodontalis*) have been previously identified qualitatively in AF collected under sterile conditions^22–24,26,36^; among these, *Ureaplasma* spp. is frequently detected in spontaneous preterm birth^36,39^. Moreover, lipoprotein multiple-banded antigen from *U. parvum* has been shown to cause preterm birth in experimental animals^46^, supporting the correlation between *Ureaplasma* presence and pregnancy outcome.

In the present study, we used samples from two distant hospitals, which were sequenced twice, to avoid regional and experimental bias. For the five most dominant species (*U. parvum*, *S. agalactiae*, *G. vaginalis*, *S. anginosus* and *S. sanguinegens*) in Stage III, the data for the two institutions corresponded well. In our previous study, we had confirmed a relationship between placental inflammation (Blanc’s classification) and the 11 species defining miCAM^36^. In seven out of 10 cases (70%) with chorioamnionitis (Stage III), at least one of these species were dominantly detected; in two cases, different species, but from the same genera as those of the 11 dominant species, were detected; and in one case, no bacteria were detected^36^. These results were remarkably consistent with our current results. Moreover, in five major reports on the AF in cases of preterm birth^22–24,26,36^, in at least 70% of all cases, at least one of the 10 most dominant genera in Stage III (*Ureaplasma*, *Streptococcus*, *Gardnerella*, *Sneathia*, *Eikenella*, *Prevotella*, *Lactobacillus*, *Bacteroides*, *Porphyromonas*, *Mycoplasma*) was qualitatively detected. Thus, our results can be regarded reliable.

The seven most dominant species in Blank (*L. sphaericus*, *Delftia* sp. Cs1–4, *Pseudomonas* sp. TJI-51, *E. coli*, *Acinetobacter* sp. WC-743, *P. saccharophila*, and *P. acnes*) were estimated to originate mainly from contamination during library preparation. Of the seven most dominant species in Blank, six genera (*Delftia*, *Pseudomonas*, *Escherichia*, *Acinetobacter*, *Pelomonas*, and *Propionibacterium*) have been previously reported as common contaminants^47^. *L. sphaericus* is primarily observed in mosquito larvae; it is a spore-forming bacterium resistant to heat and ultraviolet radiation, and common in aquatic environments^48–51^; therefore, trace amounts of DNA may have contaminated reagents, tubes, or instrumentation. The low abundance and high α-diversity (more complex composition) of the seven species were consistently demonstrated in all samples dominated by these species, which is in agreement with previous reports.

For a long time, attempts have been made to diagnose chorioamnionitis and intrauterine infection prior to delivery^19,20,52–56^. Although high-accuracy diagnostic biomarkers have been reported^53^, no diagnostic standards have been established^19,55^ because of significantly overlapping confidence intervals and inconsistent associations between preterm birth and placental microbiome^1,37,38,55^. In the present study, we demonstrated that it is possible to diagnose chorioamnionitis with a high level of accuracy according to miCAM defined by metagenomic sequence profiles and 16S rDNA copy numbers in the AF. Therefore, miCAM can be used to assess the state of intrauterine infection during pregnancy, which would help in the management of cases with high risk of preterm birth.

The quantification of 16S rDNA by dPCR has been recently reported useful for evaluating the prevalence of low-abundance bacteria^57,58^. We discovered, with high reproducibility, that when placental inflammation was mild (Stage ≤ I), microbial abundance in the AF was as low as in the early second trimester, even in preterm birth (Fig. 1).

Contamination of laboratory-grade water, PCR reagents, and DNA extraction kits can potentially significantly affect structural analysis of microbiome with low abundance^47,59^. Therefore, in this study, we made every effort to operate under strictly sterile conditions. However, complete prevention of contamination is considered impossible^47^. Therefore, we used a blank control (laboratory-grade water) in DNA extraction and library preparation, which, consistent with previous data^58^, showed an extremely low presence of 16S rDNA (1–10 copies/μL). The blank samples were used to document bacterial sequences introduced during sample processing, which were excluded from the miCAM-defining species.

This study had some limitations. First, sample selection had a bias. The analysed AF specimens were obtained at caesarean section or were left-overs from clinical testing by amniocentesis^22^, which is an invasive procedure resulting in miscarriage or preterm birth in approximately 0.1–2% of cases^60–63^, and is not performed for all pregnancies. Therefore, the retrospective case-control study design made it difficult to achieve ideal control of patient characteristics, as evidenced by significant differences between groups in multigravida, preterm premature rupture of membranes, antibiotic administration before amniocentesis, caesarean section, and gestational age at amniocentesis. While there is a possibility that maternal use of antibiotics before amniocentesis influenced the results of metagenomic analysis, we considered it to be negligible; data of all of the 21 samples (A2, 10, 15, 18, 27; B6, 7, 14, 21–26; C6, 10, 12, 16–19) from patients that had not used antibiotics before amniocentesis, were in line with the findings based on the other samples. Second, 16S rDNA sequencing is inferior to whole-genome shotgun sequencing in terms of bacterial quantification potential, because 16S rDNA sequencing has a PCR bias. However, samples with low microbial abundance are contaminated with human DNA in typical DNA extraction methods, leading to enormous whole-genome shotgun sequencing costs; therefore, a method not susceptible to the effects of human DNA should be developed. Third, metagenomic analysis cannot distinguish between live and dead bacteria, and microbial profiles in antibiotic-treated patients may not be objective.

In the present study, we comprehensively and quantitatively analysed the microbiome of the AF and, by examining its association with the degree of placental inflammation, identified bacteria significantly associated with chorioamnionitis. Further studies should focus on achieving higher diagnostic sensitivity and specificity and on developing non-invasive testing methods, which would contribute to timely diagnosis and improve perinatal outcome.

## Materials and Methods

### Study design

In total, 8,172 births occurred at the Center for Maternal, Fetal, and Neonatal Medicine, Fukuoka University Hospital and at the National Hospital Organization Saga Hospital, between August 2009 and April 2017. Placental pathology examination was conducted in 4,373 cases; among them, amniocentesis was performed for 183 patients who provided informed consent for study participation, and left-over AF samples were cryopreserved. Moreover, 10 samples were added which were obtained at caesarean section within the same period under absolutely sterile condition. To minimize bias, we established the following exclusion criteria: multiple pregnancies and amniocentesis in the early second trimester. As a result, 79 patients who passed the exclusion criteria and for whom ≥ 3 mL AF was available by amniocentesis or caesarean section were included in the study.

Patients were divided into three groups based on Blanc’s classification of placental inflammation severity^33^: Stage III (n = 32), Stage II (n = 27), and Stage 0-I (n = 20). We also established two control groups to ensure accuracy of data assessment considering the extremely low amounts of microbial DNA that can contaminate samples at any point from sample collection to sequencing. In the early second trimester (16.1 ± 0.6 weeks; mean ± S.D.), amniocentesis was performed for foetal genetic testing, and AF samples from normal pregnancies were considered as a control group (Normal AF; n = 18). Additionally, we used blank controls consisting of laboratory-grade water (Blank; n = 24) during DNA extraction and sequencing library preparation.

All methods were performed in accordance with the STARD guidelines and regulations for reporting diagnostic accuracy studies. The study was approved by the review boards of the Fukuoka University Hospital, National Hospital Organization Saga Hospital and the National Research Institute for Child Health and Development (protocol numbers 15-2-08, 23–4, and 699, respectively). Informed consent was obtained from all participants, who were explained the potential risks, including accidental leaks of personal information and project data, prior to the study. For patients who wished to withdraw content, we were able to dispose of the remaining samples, extracted DNA, and all project data at any time; however, we could not delete metagenomic sequence data which had been made publicly available through an open-access database.

### Diagnostic criteria

Histological chorioamnionitis was defined as the presence of acute inflammatory lesions of the chorion or amnion according to Blanc’s criteria^33^: stage I (sub-chorionitis): patchy or diffused accumulation of neutrophils within the sub-chorionic plate or decidua; stage II (chorionitis): more than a few scattered neutrophilic infiltrations in the chorionic plate or membranous chorionic connective tissue; and stage III (chorioamnionitis): neutrophilic infiltrates reaching sub-amniotic connective tissue and the amniotic epithelium. Funisitis was defined as neutrophilic infiltration in the umbilical vein wall or Wharton’s jelly.

### Sample collection and DNA extraction

AF samples were obtained by transabdominal ultrasound-guided amniocentesis performed percutaneously or at caesarean section under sterile conditions. The AF samples were collected in sterile tubes and preserved at 4 °C; leftover samples not needed for testing were transported on ice within 24 h to a neighbouring laboratory. To minimize changes in bacterial composition, all laboratory procedures were performed rapidly on ice. Samples were centrifuged at low speed (1,450 × *g* at 4 °C for 10 min) as previously described^22,24^; the supernatant was rapidly frozen in liquid nitrogen and preserved at −80 °C until DNA extraction.

Samples were thawed and lysed using Pathogen Lysis Tubes L (Qiagen, Hilden, Germany), and DNA was extracted using the QIAamp UCP Pathogen Mini Kit (Qiagen) according to the manufacturer’s protocol, at the National Research Institute for Child Health and Development and Fukuoka University.

### Absolute quantification of 16S rDNA

dPCR was conducted with EvaGreen dye, using universal primers (27Fmod and 338R) for 16S rDNA sequencing, as previously described^34,35,58^. DNA (1 μL) was diluted in 19 μL of Bio-Rad QX200 reagents (Bio-Rad, Hercules, CA, USA), and each sample was then partitioned into approximately 20,000 droplets using the QX200 Droplet Generator (Bio-Rad). PCR was performed in a 96-well plate using the following cycling conditions according to the manufacturer’s protocol: 5 min at 95 °C, 40 cycles of 30 s at 95 °C and 1 min at 60 °C, then 5 min at 4 °C and 5 min at 90 °C; the temperature was then decreased to 4 °C at a ramp rate of 2 °C/s. Fluorescence was detected using the QX200 Droplet Reader (Bio-Rad) and analysed using the Bio-Rad QuantaSoft software. Copy number of 16S rDNA per 1 mL of the sample was then calculated.

### Sequencing of 16S rDNA amplicons

The same universal primers (27Fmod and 338R) were used for PCR amplification of the variable region (V1–2) of the 16S rRNA gene. A 16S Metagenomic Sequencing Library was prepared according to the Illumina protocol (16S Metagenomic Sequencing Library Preparation, Part # 15044223 Rev. A; Illumina, San Diego, CA, USA). PCR was performed using the KAPA HiFi HotStart Ready Mix (Kapa Biosystems, Boston, MA, USA) for 25 amplification cycles at 60 °C as an annealing temperature according to the manufacturer’s recommendation. The amplified products were purified using the Agencourt AMPure XP Kit (Beckman Coulter, Tokyo, Japan) and tagged with indexes in eight cycles using the Nextera XT Index Kit (Illumina). Amplicons were quantified using the Agilent 2200 TapeStation (Agilent Technologies, Santa Clara, CA, USA) or the Agilent 2100 Bioanalyzer (Agilent Technologies) and thoroughly mixed to achieve homogeneity. Then, size selection for next-generation sequencing was performed using Pippin Prep (Sage Science, Beverly, MA, USA), with approximately 300–600 bp of the mixed library as a target. MiSeq sequencing (paired-end, 300 bp) was conducted using MiSeq Reagent Kit v3 (600-cycle format; Illumina) mixed with 20% of PhiX Control Kit v3 (Illumina) according to the manufacturer’s protocol.

### Analysis of sequencing data

Sequencing data was analysed as previously described^34–36^. Two paired-end reads were merged using the fastq-join program based on overlapping sequences. Low-quality sequence reads (quality value < 25) and suspected chimeric reads (BLAST match length of <90% with reference sequences in the databases [Ribosomal Database Project v. 10.27 and/or in-house 16S sequenced database in Tokyo University]) were filtered out (Table S7). Following adapter sequence trimming, 1,300 reads were randomly selected. Using a 97% pairwise-identity cut-off in the UCLUST program^64^ version 5.2.32 (http://www.drive5.com/), the selected reads were clustered into OTUs.

Taxonomic assignment for each OTU was done by similarity searching against the above-mentioned databases using the GLSEARCH program (data provided in Supplementary Dataset 1). For the assignment at the phylum, genus, and species levels, sequence similarity thresholds of 70%, 94% and 97%, respectively, were applied.

UniFrac distance was used to assess dissimilarity (distance) between each sample pair^65^, and a 3D PCoA model was plotted according to UniFrac distances using the R package.

### Statistical analysis

Because of the relatively low sample number, we calculated exact significance probabilities (two-tailed) as *P*-values using the Mann–Whitney test for continuous variables and Fisher’s exact test for categorical variables. To assess diagnostic accuracy, we constructed ROC curves and calculated the AUC. These analyses were performed using SPSS version 16.0J for Windows Base System SC (SPSS Japan, Tokyo, Japan). For comparison of bacterial composition between groups, we used vegan package in R to calculate R^2^ and *P*-values in PERMANOVA. Differences at *P* < 0.05 were considered statistically significant.

### Data and material availability

The 16S sequencing data generated in the present study have been deposited in DDBJ Sequence Read Archive (DRA) (accession number DRA005144).

## Electronic supplementary material

Supplementary Information

Supplementary Dataset 1
