# Supplementary material for: Microbiome profile of the amniotic fluid as a predictive biomarker of perinatal outcome
Source: Sci Rep. 2017 Sep 22;7:12171. doi: 10.1038/s41598-017-11699-8 (PMC5610236; doi:10.1038/s41598-017-11699-8)
Supplement: Supplementary file 1 — Supplementary Information [file 41598_2017_11699_MOESM1_ESM.pdf]

Supplementary Information

Microbiome profile of the amniotic fluid as a predictive biomarker of perinatal outcome

Daichi Urushiyama[1,2], Wataru Suda[3,4], Eriko Ohnishi[1], Ryota Araki[2], Chihiro Kiyoshima[2], Masamitsu Kurakazu[2], Ayako Sanui[5], Fusanori Yotsumoto[2], Masaharu Murata[5], Kazuki Nabeshima[6], Shin'ichiro Yasunaga[7], Shigeru Saito[8], Makoto Nomiyama[9], Masahira Hattori[3,10], Shingo Miyamoto[2], Kenichiro Hata[1*]

[1]Department of Maternal-Fetal Biology, National Research Institute for Child Health and Development, Tokyo 157-8535, Japan

[2]Department of Obstetrics and Gynecology, Faculty of Medicine, Fukuoka University, Fukuoka 814-0180, Japan

[3]Department of Computational Biology, Graduate School of Frontier Sciences, The University of Tokyo, Chiba 277-8561, Japan

[4]Department of Microbiology and Immunology, Keio University School of Medicine, Tokyo 160-0016, Japan

[5]Center for Maternal, Fetal and Neonatal Medicine, Fukuoka University Hospital, Fukuoka, 814-0180, Japan

[6]Department of Pathology, Fukuoka University School of Medicine and Hospital, Fukuoka 814-0180, Japan

[7]Department of Biochemistry, Faculty of Medicine, Fukuoka University, Fukuoka 814-0180, Japan

[8]Department of Obstetrics and Gynecology, University of Toyama, Toyama 930-0194, Japan

[9]Department of Obstetrics and Gynecology, National Hospital Organization Saga Hospital, Saga 849-8577, Japan

[10]Cooperative Major in Advanced Health Science, Graduate School of Advanced Science and Engineering, Waseda University, Tokyo 169-8555, Japan

[*]**Corresponding Author:** Kenichiro Hata, MD, PhD

Director,

Department of Maternal-Fetal Biology

National Research Institute for Child Health and Development

2-10-1 Okura, Setagaya, Tokyo 157-8535, Japan

Tel: +81-3-3416-0181

Fax: +81-3-3417-2864

E-mail: hata-k@ncchd.go.jp

Supplementary Materials and Methods

Study design

Because amniocentesis is an invasive procedure that can result in premature rupture of membranes, iatrogenic preterm birth, and other negative consequences, we used leftover samples of the amniotic fluid (AF) to the full extent permitted by major Japanese guidelines.

To minimize bias, cases with miscarriage, full-term (37 weeks or later) admission, or foetuses with abnormalities (trisomy 21, deformity, tumours) were excluded in assessment of maternal prognostic parameters. Cases with foetuses with major abnormalities or full-term delivery were excluded in assessment of foetal prognostic parameters.

Diagnostic criteria

According to Lencki et al.[20], clinical chorioamnionitis was diagnosed if patients demonstrated fever ≥38°C with no evident infection and at least one of the following signs or symptoms: maternal pulse ≥100 beats/min, uterine tenderness, malodorous vaginal discharge, or maternal white blood cell count ≥15,000/mm^3. Premature rupture of the membranes was considered to have occurred if we observed evident outflow of weakly alkaline amniotic fluid or detected insulin-like growth factor-binding protein 1 (IGFBP-1) in vaginal discharge.

Sequencing of 16S rDNA amplicons

All sequence data were obtained in two sequencing rounds. Forty-five samples (Fukuoka University Hospital) were sequenced first, 34 (Fukuoka University Hospital, 12; Saga Hospital, 22) samples were sequenced in a second round. To consider contamination in DNA extraction

and library preparation, 32 (Normal AF, 18; Blank, 14) and 30 (Normal AF, 10; Blank, 20) samples were sequenced in the first and second sequencing round, respectively. Moreover, 10 samples in each group were sequenced twice (D1-10, N1-10; ”-2” added in sample number were samples sequenced at second time.)

Supplementary Figures

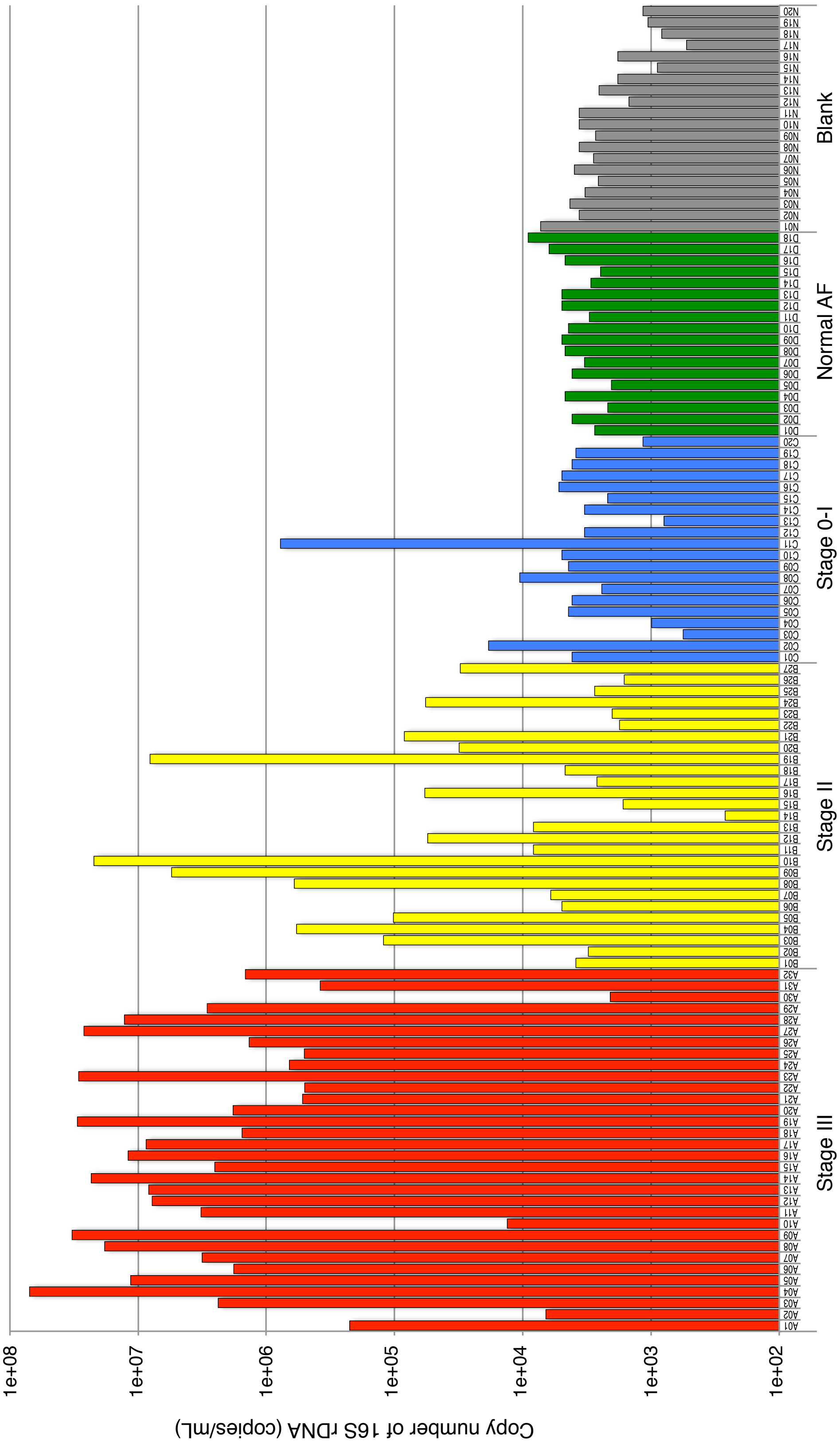

Fig. S1. Microbial abundance in each sample. Microbial load was assessed based on 16S rDNA copy numbers per 1 mL AF using dPCR with universal primers 27Fmod and 338R and EvaGreen dye.

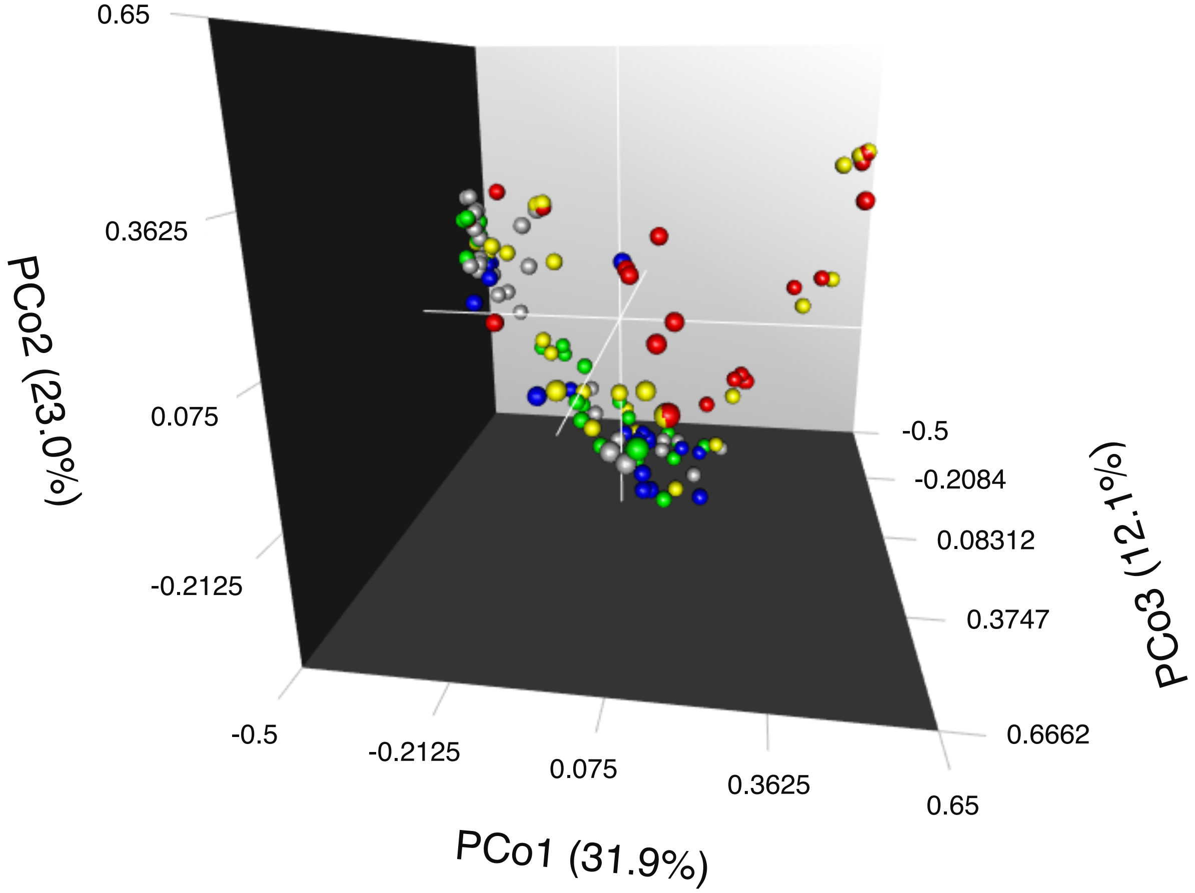

Fig. S2. 3D-PCoA based on weighted UniFrac distances. Multidimensional composition of each group was determined based on matrix data for weighted UniFrac distance. Clustering of Stage III (red) samples differed from that of Stage 0-I (blue)/Normal AF (green)/Blank (grey); Stage II (yellow) was scattered between the two clusters. 3D PCoA was conducted with R.

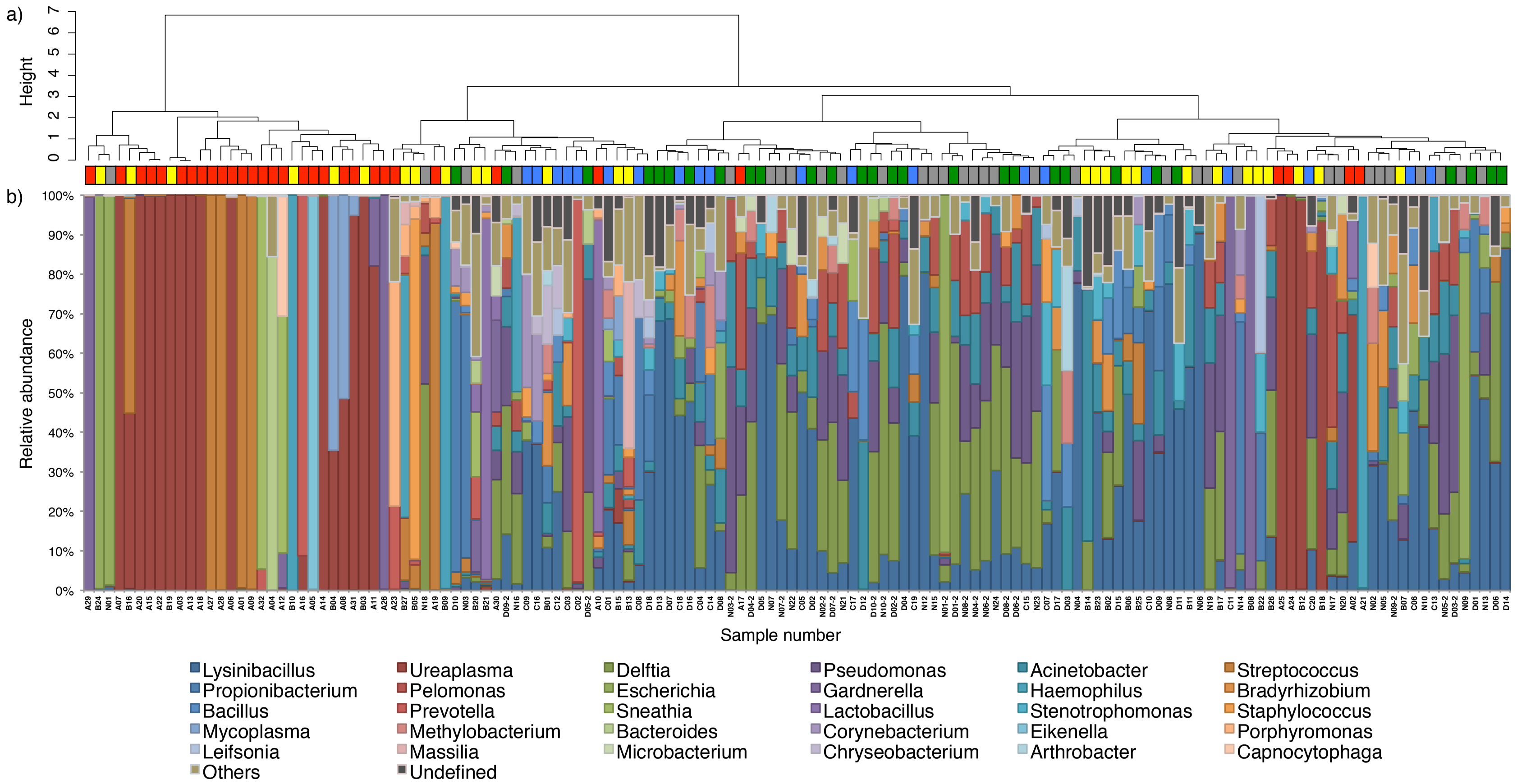
a)
Height
0 1 2 3 4 5 6 7
b)
Relative abundance
0% 10% 20% 30% 40% 50% 60% 70% 80% 90% 100%
Sample number
Lysinibacillus
Propionibacterium
Bacillus
Mycoplasma
Leifsonia
Others
Ureaplasma
Pelomonas
Prevotella
Methylobacterium
Massilia
Undefined
Delftia
Escherichia
Sneathia
Bacteroides
Microbacterium
Pseudomonas
Gardnerella
Lactobacillus
Corynebacterium
Chryseobacterium
Acinetobacter
Haemophilus
Stenotrophomonas
Eikenella
Arthrobacter
Streptococcus
Bradyrhizobium
Staphylococcus
Porphyromonas
Capnocytophaga

Fig. S3. Relative abundances of different bacterial genera in each sample. Sequences were clustered into operational taxonomic units (OTUs) with a 94% identity threshold and taxonomic assignments were performed by similarity search against the standard database. The samples were rearranged by hierarchical cluster analysis using Ward's method based on un-weighted UniFrac distances. Only major genera with top 30 are displayed. Many samples in Stage 0-I/Normal AF/Blank demonstrated a relatively high species richness indicating complex compositions. In contrast, the numbers of species in Stage III and some samples of Stage II were extremely low.

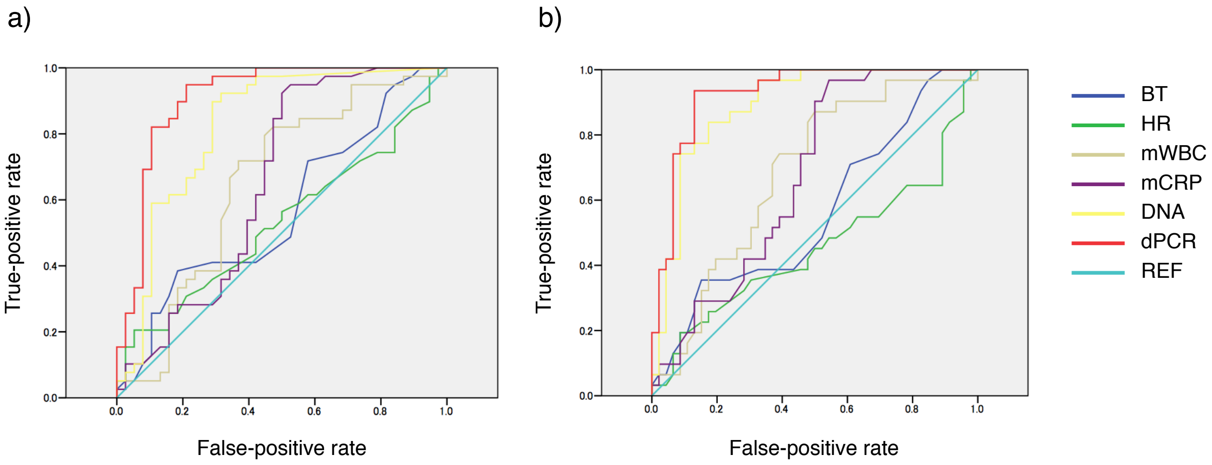

Fig. S4. Diagnostic and predictive accuracy of clinical and laboratory data, DNA content, and 16S rDNA copy number for miCAM and chorioamnionitis. Using clinical and laboratory data extracted from medical records, and DNA content and results of digital (d) PCR obtained in this study, we calculated the area under the curve (AUC) and the Youden index for the receiver operating characteristic curve (ROC), cutoff value, and detection sensitivity and specificity. Compared to body temperature (BT), heart rate (HR), white blood cell (WBC) counts, and C-reactive protein (CRP) values in maternal peripheral blood, DNA amount or 16S rDNA copy number per 1 mL of AF were more accurate parameters for the diagnosis of both miCAM and chorioamnionitis; between these, 16S rDNA copy number demonstrated the highest diagnostic accuracy.

Supplementary Tables

Table S1. Group definitions.

**Name**	**Definition (Blanc's classification)**	**Symbol**	**Sample number**
Stage III	Chorioamnionitis (Stage III)	A	A1-32
Stage II	Chorionitis (Stage II)	B	B1-27
Stage 0-I	Sub-chorionitis (Stage I) or no neutrophil infiltration	C	C1-20
Normal AF	Control of amniotic fluid in normal pregnancy	D	D1-18
Blank	Blank control	N	N1-24

Table S2. Demographic and clinical characteristics of patients in each group.

	(A) Stage III	(B) Stage II	(C) Stage 0-I	*P*-value (A vs. C)	*P*-value (B vs. C)	*P*-value (A vs. B)
**Maternal**	**(n = 32)**	**(n = 27)**	**(n = 20)**			
Age (years) †	33.0 (30–38)	31.0 (25–36)	32.0 (30–34)	0.203	0.919	0.154
Multigravida §	23 (71.9)	13 (48.1)	5 (25.0)	0.002	0.137	0.107
History of smoking §	5 (15.6)	3 (11.1)	1 (5.0)	0.387	0.626	0.715
History of cervical operation §	6 (18.8)	3 (11.1)	1 (5.0)	0.228	0.626	0.488
History of arterial abortion §	9 (28.1)	3 (11.1)	2 (10.0)	0.170	1.000	0.193
History of miscarriage or preterm birth §	6 (18.8)	5 (18.5)	0 (0.0)	0.228	0.221	1.000
History of caesarean section §	4 (12.5)	5 (18.5)	3 (15.0)	1.000	1.000	0.719
Hydramnion with preterm labor §	0 (0.0)	4 (14.8)	3 (15.0)	0.052	1.000	0.039
Preterm premature rupture of membranes §	17 (53.1)	5 (18.5)	4 (20.0)	0.022	1.000	0.008
Antibiotics before amniocentesis §	27 (84.4)	18 (66.7)	10 (50.0)	0.012	0.368	0.134

Drugs for tocolysis §	23 (71.9)	19 (70.4)	10 (50.0)	0.144	0.226	1.000
Antenatal corticosteroid before amniocentesis §	22 (68.8)	12 (44.4)	9 (45.0)	0.146	1.000	0.071
Caesarean section §	13 (40.6)	14 (51.9)	17 (85.0)	0.002	0.029	0.440
Gestational age at amniocentesis (weeks) †	28.71 (26.4–31.8)	31.14 (26.1–33.8)	32.36 (29.6–35.3)	0.009	0.182	0.230
Body temperature at amniocentesis (°C) †	36.80 (36.6–37.4)	36.90 (36.7–37.2)	36.85 (36.4–37.0)	0.143	0.082	0.859
Heart rate at amniocentesis (beats/min) †	85.0 (74–104)	96.0 (84–100)	81.0 (76–98)	0.605	0.054	0.154
WBC count in maternal peripheral blood at amniocentesis (cells/μL) †	1.42×10^4 (1.2×10^4–2.0×10^4)	1.17×10^4 (7.2×10^3–1.9×10^4)	1.07×10^4 (8.6×10^3–1.2×10^4)	0.002	0.650	0.086
CRP in maternal peripheral blood at amniocentesis (mg/dL) ‡	2.40 (1.0–5.0; 31)	1.90 (0.5–4.0; 27)	0.30 (0.1–1.7; 19)	0.001	0.040	0.161
Clinical chorioamnionitis at	4 (12.5)	3 (11.1)	1 (5.0)	0.634	0.626	1.000

amniocentesis §						
WBC in amniotic fluid (cells/μL) ‡	1,091.0 (573–1880; 11)	1,320.0 (16–3985; 7)	17.5 (11–24; 2)	0.026	0.639	0.840
Glucose in amniotic fluid (mg/dL) ‡	4.0 (1–14; 24)	8.5 (2–20; 12)	9.5 (8–31; 4)	0.285	0.706	0.479
Microscopic test by Gram staining method ¶	10/26 (38.5)	3/13 (23.1)	2/5 (40.0)	1.000	0.583	0.477
Positive culture test of amniotic fluid ¶	11/26 (42.3)	4/13 (30.8)	2/5 (40.0)	1.000	1.000	0.728
Funisitis of umbilical cord ¶	19/31 (61.3)	4/27 (14.8)	2/20 (10.0)	<0.001	1.000	<0.001
Extended days of hospital stay from admission to birth (days) ‡	3.5 (1–9; 30)	9.0 (2–23; 25)	22.0 (6–45; 17)	0.001	0.017	0.151
Majour complications after birth §	2 (6.3)	2 (7.4)	2 (10.0)	0.634	1.000	1.000
Days of hospital stay after birth (days) ‡	6.0 (4–7; 32)	5.0 (4–6; 27)	5.0 (5–8; 19)	0.642	0.224	0.513
**Neonatal**	**(n = 30)**	**(n = 20)**	**(n = 12)**			
Gestational age at birth (weeks) †	28.93 (26.9–32.4)	30.71 (26.0–32.8)	31.14 (26.9–32.8)	0.606	0.723	0.871
Neonatal body weight at birth (g) †	1,146 (990-	1,581 (833–	1,422 (905–	0.790	0.893	0.495

	1,663)	1,989)	1,862)			
Apgar score 1 min †	7.0 (5–8)	6.0 (5-8)	7.0 (6–8)	0.719	0.863	0.799
Apgar score 5 min †	9.0 (8–9)	8.0 (7-9)	9.0 (8–9)	0.902	0.215	0.092
Umbilical arterial pH at birth ‡	7.350 (7.30–7.39; 29)	7.343 (7.31–7.40; 20)	7.347 (7.31–7.37; 12)	0.805	0.752	0.705
WBC count after birth (cells/μL) ‡	1.65×10^4 (1.0×10^4–2.0×10^4; 25)	1.17×10^4 (9.4×10^3–1.6×10^4; 18)	8.20×10^3 (6.3×10^3–1.2×10^4; 10)	0.008	0.111	0.160
CRP after birth (mg/dL) ‡	0.20 (0.0–0.4; 25)	0.00 (0.0–0.1; 18)	0.00 (0.0–0.0; 10)	0.016	0.453	0.041
IgM after birth (mg/dL) ‡	12.0 (7–15; 16)	9.0 (7–10; 14)	5.0 (4–8; 9)	0.005	0.025	0.202
Antibiotics for newborn ¶	13/16 (81.3)	5/15 (33.3)	1/9 (11.1)	0.002	0.351	0.011
Sepsis ¶	3/25 (12.0)	3/18 (16.7)	1/10 (10.0)	1.000	1.000	0.683

†Data shown as median (interquartile range).

‡Data shown as median (interquartile range; n).

§Data shown as n (%).

¶Data shown as n/N (%).

WBC, white blood cell; CRP, C-reactive protein; IgM, immunogloblin M.

Table S3. Statistical analysis of UniFrac distance in each group.

Category	No. of subjects	PERMANOVA			
		Un-weigthed UniFrac		Weigthed UniFrac	
		R^2	*P*-value	R^2	*P*-value
A vs B	A: 32<br>B: 27	0.04874	**0.000999**	0.06290	**0.003996**
A vs C	A: 32<br>C: 20	0.11023	**0.000999**	0.18466	**0.000999**
A vs D	A: 32<br>D: 28	0.12949	**0.000999**	0.23891	**0.000999**
A vs N	A: 32<br>N: 33	0.12858	**0.000999**	0.23777	**0.000999**
B vs C	B: 27<br>C: 20	0.03247	**0.036960**	0.06248	**0.004995**
B vs D	B: 27<br>D: 28	0.03815	**0.003996**	0.06953	**0.002997**

B vs N	B: 27<br>N: 33	0.04993	**0.000999**	0.09704	**0.000999**
C vs D	C: 20<br>D: 28	0.02564	0.197800	0.03038	0.159800
C vs N	C: 20<br>N: 33	0.03949	**0.005994**	0.08095	**0.006993**
D vs N	D: 28<br>N: 33	0.02578	0.05894	0.02967	0.13090

Table S4. Distribution of patients between miCAM and non-miCAM subgroups.

Name	Definition	miCAM (n = 40)	non-miCAM (n = 39)
Stage III	Chorioamnionitis	30	2
Stage II	Chorionitis	8	19
Stage 0-I	Sub-chorionitis or no neutrophil infiltration	2	18

Table S5. Clinical characteristics of miCAM and non-miCAM patients.

	miCAM	non-miCAM	*P*-value (miCAM vs. non-miCAM)
**Histological diagnosis**	**(n = 40)**	**(n = 39)**	
Chorioamnionitis (Blanc's stage III) §	30 (75.0)	2 (5.1)	<0.001
Chorionitis (Blanc's stage ≥ II) §	38 (95.0)	21 (53.8)	<0.001
Funisitis ¶	20/39 (51.3)	5/39 (12.8)	0.001
**Maternal data**	**(n = 40)**	**(n = 39)**	
Age (years) †	33.0 (31–37)	31.0 (25–35)	0.016
Multigravida §	29 (72.5)	12 (30.8)	<0.001
History of smoking §	6 (15.0)	3 (7.7)	0.481
History of cervical operation §	6 (15.0)	4 (10.3)	0.737
History of arterial abortion §	11 (27.5)	3 (7.7)	0.037
History of miscarriage or preterm birth §	9 (22.5)	3 (7.7)	0.115

History of caesarean section §	8 (20.0)	4 (10.3)	0.348
Hydramnion with preterm labor §	0 (0.0)	7 (17.9)	0.005
Preterm premature rupture of membranes §	17 (42.5)	9 (23.1)	0.094
Antibiotics before amniocentesis §	35 (87.5)	20 (51.3)	0.001
Drugs for tocolysis §	30 (75.0)	22 (56.4)	0.100
Antenatal corticosteroid before amniocentesis §	26 (65.0)	17 (43.6)	0.072
Caesarean section §	20 (50.0)	24 (61.5)	0.367
Gestational age at amniocentesis (weeks) †	28.71 (25.9–32.5)	32.00 (28.4–35.4)	0.023
Body temperature at amniocentesis(°C) †	36.80 (36.6–37.4)	36.90 (36.6–37.1)	0.441
Heart rate at amniocentesis (beats/min) †	89.0 (76–104)	86.0 (78–99)	0.617
WBC count in maternal peripheral blood at amniocentesis (cells/µL) †	13,850.0 (11,475–20,025)	10,400.0 (7,200–17,200)	0.017
CRP in maternal peripheral blood at amniocentesis (mg/dL) †	1.85 (0.9–4.5)	0.69 (0.1–4.0)	0.020
Clinical chorioamnionitis at amniocentesis §	6 (15.0)	2 (5.1)	0.263

WBC in amniotic fluid (cells/μL) ‡	1,200.0 (640–3,000; 13)	27.0 (5–645; 7)	0.036
Glucose in amniotic fluid (mg/dL) ‡	4.0 (1–12; 26)	11.5 (2-31; 14)	0.402
Microscopic test by Gram staining method ¶	12/29 (41.4)	3/15 (20.0)	0.195
Positive culture test of amniotic fluid ¶	14/29 (48.3)	3/15 (20.0)	0.104
Amount of DNA per 1 mL of amniotic fluid (ng) †*	294.8 (43-1,309)	8.7 (0-55)	0.001
Copy number of 16S rDNA per 1 mL of amniotic fluid (copys) †	1.67×10^6 (3.4×10^5–9.0×10^6)	4.13×10^3 (2.5×10^3–8.3×10^3)	<0.001
Extended days of hospital stay from admission to birth (days) ‡	4.0 (0–10; 38)	18.0 (4–34; 34)	<0.001
Majour complications after birth §	3 (7.5)	3 (7.7)	<0.001
Days of hospital stay after birth (days) ‡	5.0 (4-7; 40)	5.0 (4-6; 38)	0.841
**Neonatal data**	**(n = 38)**	**(n = 24)**	
Gestational age at birth (weeks) †	28.93 (26.6–32.5)	30.71 (26.9–32.5)	0.816
Neonatal body weight at birth (g) †	1,146.0 (916–1,823)	1,425.5(872–1,834)	0.827
Apgar score 1 min †	7.0 (5–8)	6.0 (5–8)	0.963

Apgar score 5 min †	9.0 (8–9)	8.5 (7–9)	0.519
Umbilical arterial pH at birth ‡	7.350 (7.31–7.40; 37)	7.338 (7.31–7.39; 24)	0.605
WBC count after birth (cells/μL) ‡	15,250.0 (10,700–20,550; 32)	9,700.0 (7,200–13,900; 21)	0.005
CRP after birth (mg/dL) ‡	0.10 (0.0–0.44; 32)	0.00 (0.0–0.0; 21)	0.044
IgM after birth(mg/dL) ‡	11.0 (6–14; 22)	8.0 (5–9; 17)	0.026
Antibiotics for newborn ¶	15/23 (65.2)	4 (23.5)	0.012
Sepsis ¶	5 (15.6)	2 (9.5)	0.690

†Data are shown as median (interquartile range).

‡Data are shown as median (interquartile range; n).

§Data are shown as n (%).

¶Data are shown as n/N (%)

*Values < 0.2 are shown as 0.

WBC, white blood cell; CRP, C-reactive protein; IgM, immunogloblin M.

Table S6. Diagnostic accuracy for miCAM and chorioamnionitis.

	AUC	Cut-off value	Youden Index	Sensitivity	Specificity
miCAM					
Body temperature at amniocentesis	0.568	37.2 °C	0.201	0.385	0.816
Heart rate at amniocentesis	0.531	111.5 beats/min	0.152	0.205	0.947
WBC count in maternal peripheral blood at amniocentesis	0.652	11,950.0 cells/mL	0.350	0.718	0.632
CRP in maternal peripheral blood at amniocentesis	0.654	0.65 mg/dL	0.423	0.923	0.500
Amount of DNA per 1 mL of amniotic fluid	0.825	29.7 ng/mL	0.608	0.897	0.711
Copy number of 16S rDNA per 1 mL of amniotic fluid	0.909	1.19×10^4 copies/mL	0.738	0.949	0.789
Chorioamnionitis (Stage III)					
Body temperature at amniocentesis	0.561	37.3 °C	0.203	0.355	0.848

Heart rate at amniocentesis	0.455	111.5 beats/min	0.107	0.194	0.913
WBC count in maternal peripheral blood at amniocentesis	0.683	11,200.0 cells/mL	0.371	0.871	0.500
CRP in maternal peripheral blood at amniocentesis	0.673	0.65 mg/dL	0.425	0.968	0.457
Amount of DNA per 1 mL of amniotic fluid	0.891	71.4. ng/mL	0.665	0.839	0.826
Copy number of 16S rDNA per 1 mL of amniotic fluid	0.926	1.73×10^4 copies/mL	0.805	0.935	0.870

AUC, area under curve; WBC, white blood cell; CRP, C-reactive protein

Table S7. Summary of sequence reads.

Group symbol	Sample number	Sample ID	Number of reads in input for quality filtering	Reads removed		
				Reads lacking primer sequences	Reads with average quality < 25	Possible chimeric reads
Stage III	A01	06G-5th-FU671AFsn	10000	61	9	35
Stage III	A02	2S02D-PFU056AFsn-1	10000	3486	2	693
Stage III	A03	2S01E-PFU010AFsn-2	10000	53	1	28
Stage III	A04	2S01G-PFU025AFsn	10000	47	3	26
Stage III	A05	02H-5th-SAGA-016AFsn	10000	51	2	14
Stage III	A06	02F-5th-SAGA-014AFsn	10000	44	1	45
Stage III	A07	2S01C-FU090AFsn-1	10000	42	2	25
Stage III	A08	2S01A-PFU022AFsn-1	10000	45	2	58
Stage III	A09	02E-5th-SAGA-013AFsn	10000	61	8	252
Stage III	A10	2S02C-PFU082AFsn-1	10000	3033	3	3222

Stage III	A11	02B-5th-SAGA-010AFsn	10000	53	5	82
Stage III	A12	2S01H-PFU014AFsn-1	10000	37	1	26
Stage III	A13	2S01B-FU075AFsn	10000	51	2	34
Stage III	A14	02C-5th-SAGA-011AFsn	10000	45	5	39
Stage III	A15	2S11H-FU253AFsn	10000	59	3	20
Stage III	A16	02D-5th-SAGA-012AFsn	10000	40	4	21
Stage III	A17	03B-5th-SAGA-018AFsn	10000	1223	0	3
Stage III	A18	2S11F-FU191AFsn	10000	40	4	21
Stage III	A19	01G-5th-SAGA-007AFsn	10000	44	2	20
Stage III	A20	2S02A-FU021AFsn-1	10000	33	2	19
Stage III	A21	2S01F-PFU087AFsn-1	10000	380	0	428
Stage III	A22	2S01D-PFU020AFsn	10000	74	1	76
Stage III	A23	01F-5th-SAGA-006AFsn	10000	47	28	296
Stage III	A24	03C-5th-SAGA-019AFsn	10000	39	3	23
Stage III	A25	05H-5th-FU372AFsn	10000	45	6	54
Stage III	A26	01C-5th-SAGA-003AFsn	10000	62	4	41

Stage III	A27	01D-5th-SAGA-004AFsn	10000	33	9	49
Stage III	A28	06B-5th-FU619AFsn	10000	47	0	1
Stage III	A29	2S02B-PFU026AFsn-1	10000	107	0	113
Stage III	A30	05G-5th-FU345AFsn	10000	3318	0	357
Stage III	A31	03E-5th-SAGA-031AF	10000	43	3	85
Stage III	A32	03F-5th-SAGA-032AF	10000	48	4	23
Stage II	B01	2S03D-PFU080AFsn-1	20000	9910	7	4967
Stage II	B02	2S11B-FU122AFsn	30000	9804	4	16020
Stage II	B03	2S02G-FU080AFsn	10000	91	1	10
Stage II	B04	2S02H-PFU018AFsn-1	10000	61	1	7
Stage II	B05	02A-5th-SAGA-009AFsn	10000	91	13	67
Stage II	B06	2S04A-PFU009AFsn-2	10000	5419	2	501
Stage II	B07	2S04D-FU001AFsn-1	15145	8087	2	4705
Stage II	B08	2S02E-FU099AFsn-1	10000	177	0	145
Stage II	B09	01H-5th-SAGA-008AFsn	10000	35	1	16
Stage II	B10	03A-5th-SAGA-017AFsn	10000	34	1	0

Stage II	B11	2S03C-PFU115AFsn-1	30000	18494	7	6024
Stage II	B12	06C-5th-FU647AFsn	10000	87	3	31
Stage II	B13	02G-5th-SAGA-015AFsn	10000	1148	2	1281
Stage II	B14	2S04E-FU045AFsn-1	10426	7960	2	262
Stage II	B15	2S04C-PFU013AFsn-1	10000	5165	1	172
Stage II	B16	2S02F-FU109AFsn	10000	215	3	67
Stage II	B17	06A-5th-FU516AFsn	10000	1068	0	136
Stage II	B18	2S03B-FU102AFsn-1	10000	2323	1	21
Stage II	B19	2S03A-PFU029AFsn-1	10000	69	0	55
Stage II	B20	2S03F-PFU012AFsn-1	10000	452	7	342
Stage II	B21	06H-5th-FU795AFsn	8431	77	2	26
Stage II	B22	2S03E-PFU016AFsn-2	44528	39792	9	230
Stage II	B23	2S04B-PFU004AFsn3-1	10000	6483	3	48
Stage II	B24	2S03G-PFU048AFsn-1	10000	155	0	6
Stage II	B25	2S03H-PFU114AFsn-1	20000	15136	2	98
Stage II	B26	06D-5th-FU662AFsn	10000	1422	2	285

Stage II	B27	03D-5th-SAGA-020AFsn	10000	223	5	71
Stage 0-I	C01	2S06A-PFU023AFsn-1	10000	3977	1	555
Stage 0-I	C02	01A-5th-SAGA-001AFsn	10000	163	4	118
Stage 0-I	C03	06F-5th-FU674AFsn	10000	2745	3	133
Stage 0-I	C04	01B-5th-SAGA-002AFsn	10000	1793	4	2016
Stage 0-I	C05	2S05F-FU038AFsn-1	10000	6264	1	391
Stage 0-I	C06	2S05C-PFU112AFsn-1	10000	5678	1	710
Stage 0-I	C07	2S11D-FU130AFsn	10000	5904	1	430
Stage 0-I	C08	2S04F-FU107AFsn	20000	14332	3	767
Stage 0-I	C09	2S05A-PFU006AFsn-1	10000	5608	2	100
Stage 0-I	C10	2S05H-FU058AFsn-1	10000	6061	0	79
Stage 0-I	C11	01E-5th-SAGA-005AFsn	10000	60	1	11
Stage 0-I	C12	2S05D-PFU002AFsn-1	10000	6852	2	17
Stage 0-I	C13	06E-5th-FU621AFsn	10000	1963	1	93
Stage 0-I	C14	2S05E-PFU005AFsn-1	10000	5795	0	607
Stage 0-I	C15	05F-5th-FU314AFsn	10000	3066	2	25

Stage 0-I	C16	2S04H-PFU041AFsn-1	10000	5405	2	224
Stage 0-I	C17	2S05G-PFU007AFsn-1	20000	14637	3	124
Stage 0-I	C18	2S04G-PFU090AFsn-1	7423	4747	2	392
Stage 0-I	C19	2S05B-PFU091AFsn-1	10000	6217	1	730
Stage 0-I	C20	07A-5th-FU800AFsn	10000	1932	2	544
Normal AF	D01	2S06F-PFU061AFsn-2	39671	33690	3	2840
Normal AF	D01-2	10A-5th-D01-2S06F-PFU061AFsn-2	10000	2603	3	2
Normal AF	D02	2S06G-PFU101AFsn-1	10000	6805	0	72
Normal AF	D02-2	10B-5th-D02-2S06G-PFU101AFsn-1	10000	2416	3	6
Normal AF	D03	2S08D-FU057AFsn-1	12100	9819	2	108
Normal AF	D03-2	10C-5th-D03-2S08D-FU057AFsn-1	10000	2703	1	18
Normal AF	D04	2S06D-PFU047AFsn-2	10000	4270	2	285
Normal AF	D04-2	10D-5th-D04-2S06D-PFU047AFsn-2	10000	2200	2	90
Normal AF	D05	2S08A-FU031AFsn-1	20000	14046	0	50
Normal AF	D05-2	10E-5th-D05-2S08A-FU031AFsn-1	10000	1305	3	25
Normal AF	D06	2S07D-PFU092AFsn-1	18387	16954	3	69

Normal AF	D06-2	10F-5th-D06-2S07D-PFU092AFsn-1	10000	2764	1	28
Normal AF	D07	2S06E-FU081AFsn-1	10000	6130	2	40
Normal AF	D07-2	10G-5th-D07-2S06E-FU081AFsn-1	10000	2674	0	9
Normal AF	D08	2S06B-PFU052AFsn-2	20000	13791	1	285
Normal AF	D08-2	10H-5th-D08-2S06B-PFU052AFsn-2	10000	1359	3	28
Normal AF	D09	2S06H-PFU104AFsn-1	10000	6477	3	149
Normal AF	D09-2	11A-5th-D09-2S06H-PFU104AFsn-1	10000	2567	2	8
Normal AF	D10	2S07H-PFU079AFsn-1	10000	898	1	62
Normal AF	D10-2	11B-5th-D10-2S07H-PFU079AFsn-1	10000	2264	1	37
Normal AF	D11	2S06C-PFU108AFsn-1	10000	6843	1	91
Normal AF	D12	2S07F-PFU109AFsn-1	35677	32298	7	258
Normal AF	D13	2S07E-PFU105AFsn-1	10000	6315	0	31
Normal AF	D14	2S07G-PFU103AFsn-1	20000	14390	3	483
Normal AF	D15	2S08B-FU019AFsn-1	7355	5810	5	38
Normal AF	D16	2S07C-PFU102AFsn-1	10000	6606	0	175
Normal AF	D17	2S07A-PFU055AFsn-1	20000	14748	3	70

Normal AF	D18	2S07B-PFU107AFsn-1	20000	14288	3	209
Blank	N01	2S08E-NCQP141204-1	10000	606	2	0
Blank	N01-2	11C-5th-N01d-2S08E-NCQP141204-1	10000	282	1	1
Blank	N02	2S08G-NCQP141209-1	10000	5648	2	139
Blank	N02-2	11D-5th-N02d-2S08G-NCQP141209-1	10000	2627	1	6
Blank	N03	2S08H-NCQP141210-1	10000	1399	1	63
Blank	N03-2	11E-5th-N03d-2S08H-NCQP141210-1	10000	5245	1	3
Blank	N04	2S09A-NCQP141211-1	12362	8993	0	4
Blank	N04-2	11F-5th-N04d-2S09A-NCQP141211-1	10000	2853	0	20
Blank	N05	2S09B-NCQP150107-1	6411	3973	1	8
Blank	N05-2	11G-5th-N05d-2S09B-NCQP150107-1	10000	3197	0	1
Blank	N06	2S09C-NCQP150116-1	6505	4247	1	8
Blank	N06-2	11H-5th-N06d-2S09C-NCQP150116-1	10000	936	3	2
Blank	N07	2S12E-NCQP151023-1	20000	14586	1	41
Blank	N07-2	12A-5th-N07d-2S12E-NCQP151023-1	10000	1637	0	5
Blank	N08	2S09G-1NCPCR	5237	3299	2	7

Blank	N08-2	12B-5th-N08s-2S09G-1NCPCR	10000	2123	1	2
Blank	N09	2S09D-2NCPCR-1	4135	2446	0	4
Blank	N09-2	12C-5th-N09s-2S09D-2NCPCR-1	10000	1801	1	2
Blank	N10	2S09E-2NCPCR-2	5426	3973	1	11
Blank	N10-2	12D-5th-N10s-2S09E-2NCPCR-2	10000	1635	1	3
Blank	N12	2S12F-3NCPCR-1	20000	15476	2	33
Blank	N13	2S12G-3NCPCR-2	10000	6395	0	8
Blank	N14	2S12H-3NCPCR-3	10000	4634	3	14
Blank	N15	03G-5th-170607QPN1	10000	2573	2	8
Blank	N16	03H-5th-170607QPN2	10000	2249	0	155
Blank	N17	05C-5th-160815QPN1	10000	814	1	1
Blank	N18	05D-5th-160815QPN2	10000	3605	2	8
Blank	N19	07B-5th-170609QPN1	10000	2786	1	0
Blank	N20	07C-5th-170609QPN2	10000	1344	1	2
Blank	N21	12E-5th-NCPCR-1	10000	2297	2	4
Blank	N22	12F-5th-NCPCR-2	10000	4217	2	3

Blank	N23	12G-5th-NCPCR-3	10000	1960	2	2
Blank	N24	12H-5th-NCPCR-4	10000	3497	3	5
